# Prognostic Value of Serum Osteopontin in Acute Exacerbation of Idiopathic Pulmonary Fibrosis

**DOI:** 10.1155/2020/3424208

**Published:** 2020-02-10

**Authors:** Xianhua Gui, Xiaohua Qiu, Miaomiao Xie, Yaqiong Tian, Cao Min, Mei Huang, Wu Hongyan, Tingting Chen, Xin Zhang, Jingyu Chen, Mengshu Cao, Hourong Cai

**Affiliations:** ^1^Department of Respiratory and Critical Care Medicine, The Affiliated Drum Tower Hospital of Nanjing University Medical School, No. 321 Zhongshan Road, Nanjing 210008, Jiangsu, China; ^2^Department of Respiratory and Critical Care Medicine, Drum Tower Clinical Medical College of Nanjing Medical University, No. 321 Zhongshan Road, Nanjing 210008, Jiangsu, China; ^3^Department of Pathology, The Affiliated Drum Tower Hospital of Nanjing University Medical School, No. 321 Zhongshan Road, Nanjing 210008, Jiangsu, China; ^4^Department of Rheumatology and Immunology, The Affiliated Drum Tower Hospital of Nanjing University Medical School, Nanjing 210008, China; ^5^Jiangsu Key Laboratory of Organ Transplantation, Wuxi People's Hospital, Nanjing Medical University, No. 299 Qingyang Road, Wuxi 214023, Jiangsu, China

## Abstract

**Background:**

Acute exacerbation (AE) is a common cause of rapid deterioration and high mortality in idiopathic pulmonary fibrosis (IPF) patients. Osteopontin (OPN) plays an important role in IPF, but the studies about serum OPN in AE-IPF are unclear. We aimed to investigate whether OPN had a potential prognostic value in acute exacerbation and mortality in IPF.

**Methods:**

Thirty-two patients with AE-IPF, 39 with S-IPF, and 20 healthy controls were included. Serum OPN and KL-6 levels were compared between AE-IPF and S-IPF. Logistic regression analysis was applied to identify the predicted value of OPN for AE. Kaplan–Meier curves were used to display survival, and Cox proportional hazards regression was used to identify risk for mortality.

**Results:**

In AE-IPF patients, serum OPN levels were significantly higher than in S-IPF subjects (*p* < 0.001) or healthy controls (*p* < 0.001) or healthy controls (*p* < 0.001) or healthy controls (*p* < 0.001) or healthy controls (*p* < 0.001) or healthy controls (*p* < 0.001) or healthy controls (*p* < 0.001) or healthy controls (*p* < 0.001) or healthy controls (*p* < 0.001) or healthy controls (*p* < 0.001) or healthy controls (

**Conclusion:**

Elevated OPN could be a potential serum predictor for AE status and survival in IPF patients.

## 1. Introduction

Idiopathic pulmonary fibrosis (IPF) presents as a progressive deterioration of dyspnea and ultimately respiratory failure [1], characterized by epithelial cell damage and activation, fibroproliferation, and abnormal accumulation of extracellular matrix (ECM) in lung parenchyma with elusive cause [2]. The natural history of IPF is viewed as gradual progression with a slow decline in lung function over time. Recently, acute exacerbation of IPF becomes one of the most important causes of mortality. This process is known as sudden aggravation of dyspnea, new ground glass opacities on chest imaging, and diffuse alveolar damage (DAD) [3]. Statistically, the one-year and three-year incidences of AE are 14% and 20%, with more than 90% of patients needing treatment in the intensive care unit and 50% dying within a short time after diagnosis [4, 5]. AE-IPF presents a major challenge in clinical study due to its complex clinical course and the lack of effective biomarkers for relevant outcomes. Thus, there is an urgent need for circulating biomarkers in IPF to evaluate the risk of AE and predict survival accurately.

So far, the etiology and mechanisms of AE-IPF occurrence have been poorly known. More and more evidence demonstrated the importance of diffuse alveolar damage and enhanced epithelial injury and proliferation during AE in IPF patients [6]. OPN as a secreted phosphoprotein, originally detected in osteoblasts and osteoclasts, can be involved in a variety of biological processes, including cell recruitment and adhesion, immune regulation, cell survival, bone reconstruction, and wound repair [7, 8]. It is well known that OPN plays a key role in restoration of tissues, angiogenesis, and autoimmune diseases through interfering with intercellular networks. Moreover, OPN produced by bronchial epithelial cells and alveolar macrophages of the lungs [9] is also involved in pulmonary diseases, including tuberculosis and lung cancer [10, 11].

In this study, we aimed to determine whether OPN could be a biomarker of AE-IPF occurrence and mortality by comparing serum OPN levels in AE-IPF and S-IPF and by analyzing correlation between serum OPN and other variables in IPF patients.

## 2. Methods

### 2.1. Study Subjects

The study population includes 71 subjects (32 AE-IPF patients and 39 S-IPF patients), who were evaluated at Nanjing Drum Tower Hospital between January 2015 and April 2018. 20 healthy subjects from the Center of Physical Examination were included as a control group. The criteria used for enrollment of patients in this study were in accordance with the criteria of IPF and AE-IPF already published [1, 3]. The inclusion criterion for IPF was detection of a pattern of usual interstitial pneumonia on high-resolution computed tomography (HRCT). Patients with other known causes of interstitial lung disease, such as connective tissue disease with autoimmune features, domestic or occupational environmental exposure, and drug toxicity, were excluded. AE-IPF was diagnosed according to the revised diagnostic criteria described by Collard et al. in 2016. Briefly, acute exacerbation means worsening clinical symptoms within 30 days, new bilateral ground glass opacities on chest imaging, and without evidence of a definite etiology. S-IPF patients were defined as patients who had no severe symptoms of dyspnea or rapid deterioration on imaging for at least 3 months beforehand. This study was retrospectively performed and was approved by the institutional review board of the Nanjing Drum Tower Hospital affiliated to Nan Jing University. The informed consent to participate in this study and granted permission to use the serum/lung were obtained from all subjects. This study was approved by the Ethics Committee at Nanjing Drum Tower Hospital and conducted in accordance with the principles of the Declaration of Helsinki (1989).

### 2.2. Collection of Clinical Data

Clinical characteristics and vital status were derived from medical records of admission and follow-up telephone calls. Survival time was calculated from initially diagnosed time until death or censoring time.

### 2.3. Measurement of Serum OPN and KL-6

Serum samples after collection were stored at −70°C before analysis. OPN concentrations were measured by using a human OPN enzyme-linked immunosorbent assay development kit (R&D Systems, Minneapolis, MN) according to the manufacturer's instructions. The absorbance was measured at 450 nm in a microtest plate spectrophotometer, and osteopontin levels were quantified with a calibration curve using human osteopontin as a standard. Serum KL-6 levels were determined using the KL-6 kit (Fujirebio, Inc., Tokyo, Japan) on an automated immunoassay analyzer LUMIPULSE G1200 (Fujirebio, Inc., Tokyo, Japan).

### 2.4. Human Lung Specimens

Human lung tissue samples were obtained from 6 patients with IPF (UIP pattern; mean age ± SD: 62.33 ± 6.95 years; 6 males) who had undergone lung transplantation surgery at the Key Laboratory of Organ Transplantation of Wuxi People's Hospital (Wuxi, PR China). The diagnosis of IPF was verified by histological examination of the explanted lungs by pathologists. All patients fulfilled the diagnostic criteria for IPF [1]. Control lung tissue (control subjects; mean age ± SD: 64.33 ± 9.07 years; 2 females, 4 males) was collected from 6 patients undergoing surgery for cancer or pulmonary nodules at Thoracic surgery of Nanjing Drum Tower Hospital. Lung tissue samples were stored at −80°C after collection. All participants provided informed consent to participate in this study and granted permission to use the tissue. The study was approved by institutional review board.

### 2.5. Immunohistochemistry

The tissue sections were immunohistochemically stained for OPN. Briefly, sections were rehydrated and antigen retrieved; then, peroxidase was blocked in 3% H_2_O_2_ in methanol and finally was blocked with 0.25% casein in PBS. The sections were incubated with rabbit polyclonal antibody against OPN (ab8448, abcam, United Kingdom) and then were incubated with biotin-conjugated anti-rabbit IgG polyclonal antibody. Slides were counterstained with haematoxylin.

### 2.6. Statistical Analysis

Descriptive statistics of clinical characteristics are presented as mean ± standard deviation (SD). The Mann–Whitney *U* test was used to compare for variables among AE-IPF, S-IPF, and controls. Correlations between serum OPN and clinical parameters were analyzed by Spearman correlations. Logistic regression analysis was used to identify the predicted value of OPN for acute exacerbation. Cumulative survival probabilities were generated by using the Kaplan–Meier with cutoff values of OPN and KL-6 acquired from the ROC curve. The log-rank test was used to compare survival in patients. Univariate and multivariate Cox regression models were built to predict mortality. Among the statistically significant covariates in the univariate analyses, several covariates were excluded because of potential confounders and statistical limitations. The statistical analyses were performed by using SPSS Statistic (IBM Corp., Armonk, NY, USA) and Prism version 6 (GraphPad, SanDiego, CA, USA). *p* values lower than 0.05 were considered significant.

## 3. Results

### 3.1. Clinical Characteristics of Subjects

The clinical characteristics of the 71 patients and 20 control subjects are shown in Table 1. Proportions of age, gender, and smoking history did not differ between the cohorts. Both cohorts were well-matched. Most patients were old men and had a smoking history. Patients with AE-IPF had worse FVC% and DLCO% and elevated inflammatory markers such as CRP and LDH (Table 1).

### 3.2. Elevated Serum OPN during Acute Exacerbation in IPF Patients

**Figure 1:**
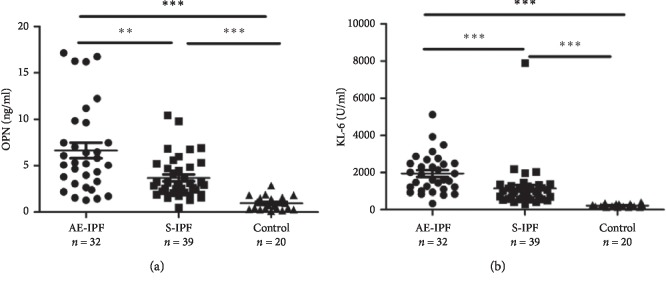
Serum OPN and KL-6 levels in patients with stable IPF and in those with AE-IPF. (a) Serum OPN concentrations were elevated significantly in AE-IPF patients when compared with S-IPF patients and healthy controls by ELISA (*p*=0.003 and *p* < 0.001, respectively). (b) Serum KL-6 levels increased obviously in AE-IPF patients compared with S-IPF patients and healthy controls (*p* < 0.001 and *p* < 0.001, respectively). AE-IPF, acute exacerbation of idiopathic pulmonary fibrosis; S-IPF, stable idiopathic pulmonary fibrosis; IPF, idiopathic pulmonary fibrosis. ^*∗*^*p* < 0.05; ^*∗∗*^*p* < 0.01; ^*∗∗∗*^*p* < 0.001 (Mann–Whitney *U* test).

Compared with controls, serum concentrations of OPN in AE-IPF patients increased obviously than in patients with S-IPF (5.24 [3.09, 9.11] versus 2.93 [2.05, 4.85] ng/ml, *p*=0.003) or healthy controls (0.71 [0.33, 1.55] ng/ml, *p* < 0.001) (Figure 1(a)). Elevated KL-6 was also observed at acute exacerbations, as compared with stable status of IPF (1744.00[1121.75, 2489.00] versus 909.00 [600.00, 1270.00] U/ml, *p* < 0.001) (Figure 1(b)).

**Figure 2:**
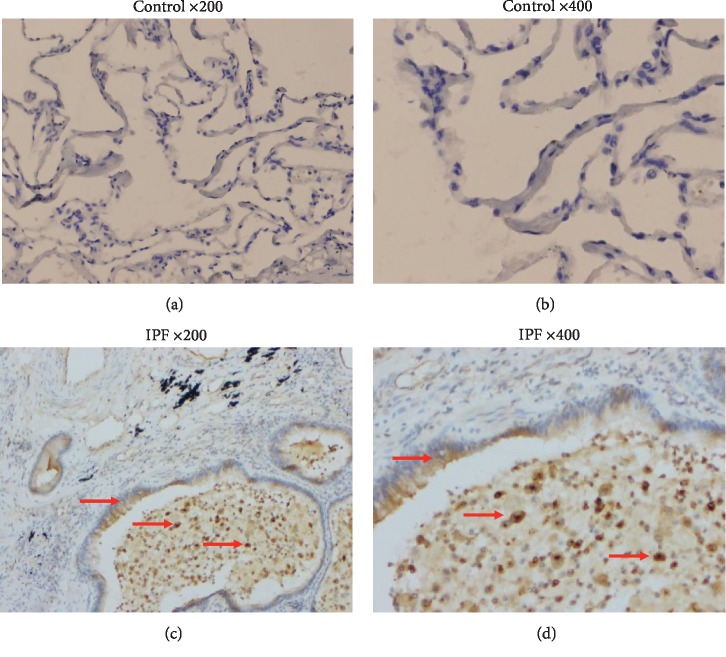
(a, b) No obvious staining for OPN was observed in the control lung. (original magnification: ×200/400). (c, d) In explanted lungs from IPF patients, the expression of OPN was found in alveolar epithelial cells lining honeycomb space and alveolar macrophages accumulating in alveolar space adjacent to fibrotic lesions (original magnification: ×200/400).

We assessed OPN expression in lung specimens from patients with IPF by using immunohistochemistry. As illustrated in Figures 2(c) and 2(d), OPN was expressed in alveolar epithelial cells lining honeycomb space and alveolar macrophages accumulating in alveolar space adjacent to fibrotic lesion. In control lung specimens, no obvious staining for OPN was observed (Figures 2(a) and 2(b)).

### 3.3. Serum OPN Concentration Correlates with CRP and LDH

**Figure 3:**
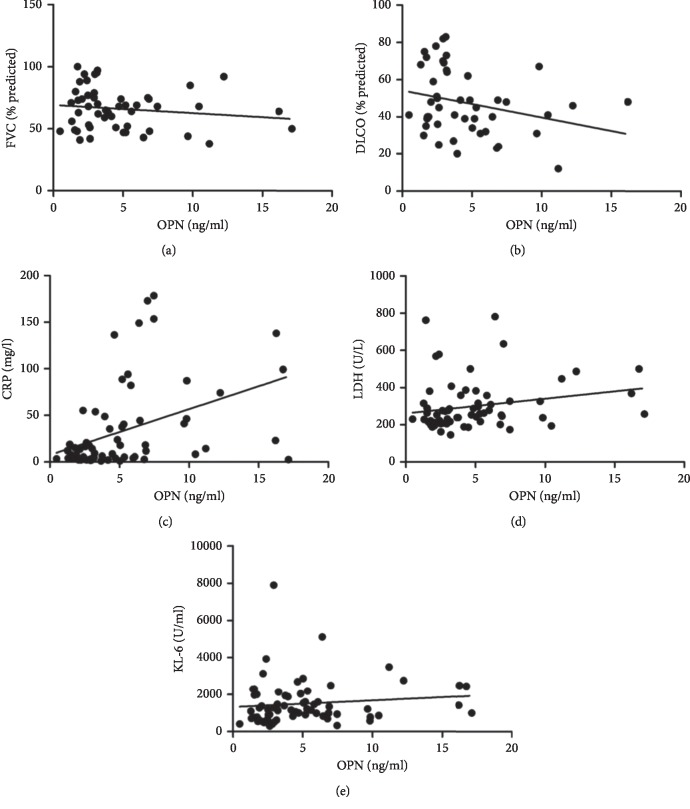
Correlations between OPN and clinical variables. (a, b, e) No correlations were noted between OPN and FVC% predicted, DLCO% predicted, and KL-6. (*r* = −0.153, *p*=0.285; *r* = −0.272, *p*=0.064; and *r* = 0.216, *p*=0.071). (c, d) Spearman correlations showed weak, yet significant correlations between OPN and inflammatory factors including CRP (*r* = 0.477, *p* < 0.001) and LDH (*r* = 0.264, *p*=0.031).

Correlation analysis with clinical variables showed that the serum concentrations of OPN in IPF patients were positively correlated with the inflammation markers such as CRP (*r* = 0.477, *p* < 0.001) and LDH (*r* = 0.264, *p*=0.031). There was no significant correlation of serum levels of OPN with KL-6 (*r* = 0.216, *p*=0.071). Furthermore, there was no significant correlation of OPN with forced vital capacity (*r* = −0.153, *p*=0.285) and diffusion capacity (*r* = −0.272, *p*=0.064) (Figure 3).

### 3.4. Predictive Value of OPN for Acute Exacerbation in IPF Patients

Logistic regression was used to determine the risk of acute exacerbation in IPF. On logistic regression, serum OPN, KL-6, CRP, LDH, and worse lung function (FVC%, DLCO% predicted) were significantly associated with a higher risk of AE in IPF, with odds ratios of 1.305 [95% CI 1.087, 1.567, *p*=0.004], 1.001 [95% CI 1.000, 1.002, *p*=0.010], 1.039[95% CI 1.013, 1.064, *p*=0.002], 1.035 [95% CI 1.017, 1.053, *p* < 0.001], 0.950 [95% CI 0.908, 0.993, *p*=0.024], and 0.929 [95% CI 0.878, 0.983, *p*=0.010], respectively (Table 2).

### 3.5. Serum OPN Predicts the Survival in IPF Patients

**Figure 4:**
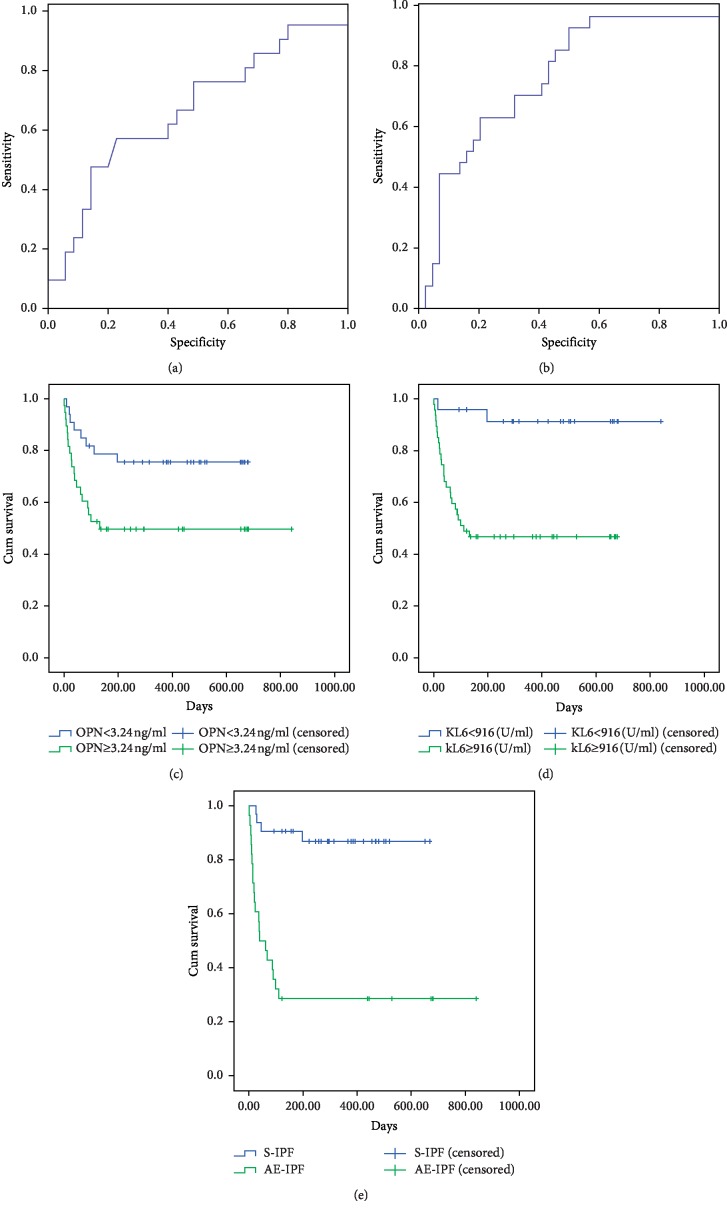
Serum OPN could predict the mortality of IPF patients. (a, b) The areas under the ROC curves of OPN and KL-6 were statistically significant in differentiating the decedent from the survivor (*p*=0.031, cutoff value 3.24 ng/ml; *p* < 0.001, cutoff value 916 U/ml, respectively). (c) Patients with serum OPN levels above 3.24 ng/ml had a shorter survival than those with OPN levels below that (*p*=0.019). (d) Patients with serum KL-6 levels above 916 U/ml had a higher mortality than those below 916 U/ml (*p* < 0.001). (e) Kaplan–Meier analyses showed mortality was significantly higher in patients with AE-IPF than in those with S-IPF by the log-rank test (*p* < 0.001).

To evaluate the potential value of serum OPN levels for predicting mortality of IPF, receiver operating characteristic (ROC) analysis was performed. The optimal cutoff value of serum OPN for predicting mortality was 3.24 ng/ml (sensitivity 57.1%, specificity 77.1%). The area under the curve (AUC) for OPN in distinguishing decedents was 0.667 (95% CI, 0.515–0.818). The largest areas under the curve were found for serum KL-6 with 0.759 (0.644–0.875). The cutoff levels set for predicting survival were 916 U/ml for KL-6 (sensitivity 92.6%, specificity 50.0%) (Figure 4(a) and 4(b)).

Kaplan–Meier curve patients with high serum OPN and KL-6 levels showed a higher mortality rate than objects with low levels in accordance with the cutoff value obtained from the ROC curve (*p*=0.019 and *p* < 0.001, respectively) (Figures 4(c) and 4(d)). Moreover, the Kaplan–Meier curve showed that AE-IPF patients had shorter survival than S-IPF (*p* < 0.001, Figure 4(e)).

To further examine the prognostic values of OPN with regard to the survival in all IPF patients, univariate Cox regression was performed (Table 3). Elevated OPN and KL-6 levels were markedly worse prognostic factors with respective hazard ratios of 1.100 (95% CI 1.006–1.202, *p*=0.036) and 1.000 (95% CI 1.000–1.000, *p*=0.007). Patient age, CRP, and LDH were also worse prognostic factors with respective hazard ratios of 1.055 [95% CI 1.007–1.105, *p*=0.025], 1.012 [95% CI 1.006–1.012, *p* < 0.001], and 1.009 [95% CI 1.006–1.019, *p* < 0.001]. Subsequently, multivariate Cox regression was performed. Several covariates were excluded for statistical limitations. We observed that OPN was also associated with the survival of IPF in multivariate Cox models, with a hazard ratio of 1.010 (95% CI 1.001–1.019, *p*=0.032). The same results were also identified for serum KL-6 (1.000 (95% CI 1.000–1.000, *p*=0.007).

## 4. Discussion

This was the first study to assess the significance of serum OPN levels in occurrence of AE and mortality in IPF patients. IPF patients had elevated serum OPN levels, especially in AE-IPF, compared with healthy controls. In addition, the enhanced expression of OPN was also found in alveolar epithelial cells and alveolar macrophages of the lung section from IPF patients. Compared with S-IPF, serum OPN levels in AE-IPF were significantly increased, which were associated with poor outcome. We found that the survival period was obviously shorter for patients with OPN levels above, compared with those below 3.24 ng/ml; moreover, OPN was a predictor of survival. Collectively, our results suggested that OPN could predict prognosis of patients with IPF.

Acute exacerbation state of IPF is an important factor contributing to IPF mortality. Unrecognized infection, diffuse alveolar damage, mechanical procedures, and secondary pulmonary hypertension have been suggested to explain acute exacerbation of IPF [12, 13], but the underlying mechanisms are poorly understood. More useful biomarkers to predict the progression and mortality of IPF disease are necessary. Blood biomarkers including SP-A [14], SP-D [15], KL-6 [16], and circulating fibrocytes [17] have been confirmed to be useful prognostically, but some of them are not easy to test. The present study showed OPN would be a new reasonable biomarker.

OPN is a secreted phosphoprotein produced by a variety of cells such as activated macrophages, vascular smooth muscle cells, and epithelial cells, mediating cell chemotaxis, adhesion, proliferation, and migration, and plays a vital role in the pathogenesis of liver and kidney fibrosis [18, 19]. Cardiac OPN expression is a strong predictor of cardiac fibrosis induced by chronic myocarditis [20]. Moreover, compared with alpha-fetoprotein (AFP), OPN as a chemoattractant for macrophages and neutrophils during injury is considered to be a better prognostic marker for early hepatocellular carcinoma (HCC) [21]. Previous studies have indicated bronchial epithelial cells and alveolar macrophages in the lungs could produce OPN. Both human and murine studies have shown that OPN plays an important role in pulmonary fibrosis. Specially, the detection of mRNA from lung biopsies of IPF shows that OPN is the most prominently expressing cytokines and expression appeared to be dependent of inflammation [22]. Furthermore, in murine models of pulmonary fibrosis, the high OPN levels accelerate the progression of fibrosis through mediating fibroblast migration, adhesion, and proliferation [23]. In addition, OPN-deficient mice exhibit reduced fibrosis characterized by a marked reduction in scar matrix [24]. On the other hand, OPN can regulate the expression of matrix metalloproteinase (MMP) to promote the accumulation of extracellular matrix (ECM) [25]. Although OPN plays a key role in IPF, there has been no report about the role of OPN involved in the development of acute exacerbations in IPF so far. We assumed that OPN could be involved in the development of acute exacerbations of IPF by mediating the inflammatory response leading to diffuse alveolar damage.

In current study, AE-IPF patients showed higher mortality than S-IPF, consistent with the reported studies [4, 26]. Elevated serum OPN concentrations were associated with acute exacerbation of IPF patients. In addition, IPF patients with serum OPN levels above the cutoff value obtained from ROC curves had higher mortality than those under cutoff value, which predicted worse outcome. The univariate and multivariate analysis also showed that OPN was a predictor of survival. In contrast, KL-6 as a classical biomarker elevating in IPF patients, especially in AE-IPF, was also proved to be useful for evaluating acute exacerbation of IPF and predicting the survival in our study. However, the test of KL-6 was not readily available, which limited its application.

Previous studies demonstrate physiologic parameters such as age, FVC%, and DLCO% have been shown to be important survival factors for IPF [27, 28]. In our study, age was predictive for mortality; an increased trend in age was associated with worse outcome. FVC% and DLCO% were closely associated with acute exacerbation occurrence. No significant difference was observed in other physiological variables.

In this study, LDH and CRP were significantly associated with acute exacerbation status and mortality. LDH is one of the earliest for the serum marker of disease activity in different forms of pulmonary fibrosis [29]. LDH, as a cytoplasmatic enzyme, indicates the cell damage or cell death, but it has high sensitivity and low specificity due to many different circumstances of cell damage caused by lack of oxygen starvation, dehydration, and infection [29]. CRP, as an acute phase protein, increases significantly by destruction in organs, infection, and inflammation. In previous studies, CRP levels have been suggested to be a prognostic factor of AE [2].

More and more studies demonstrate that acute alveolar epithelial damage and unrecognized infection play an important role in AE of lung fibrosis. This could explain why the levels of LDH and CRP increased obviously in AE-IPF. Our results indicated that elevated levels of LDH and CRP were notably associated with survival, which was consistent with previous studies, but due to their low specificity, they were difficult to be a reasonable biomarker for mortality [29].

There are several limitations in this study. Our ability to perform serial analysis of OPN was limited due to the small study population, which was understandable in consideration of the rarity of IPF. Furthermore, there were no data about OPN in the same patient experiencing the different stages of disease (e.g., stable, during and after AE), which was necessary to be a good biomarker for OPN. A comprehensive study in a larger IPF population would be helpful for further research.

## 5. Conclusions

In summary, to the best of our knowledge, this was the first study to demonstrate elevated serum OPN levels in AE-IPF patients, which was associated with acute exacerbation state and increased mortality risk. The same results were found for KL-6. More studies are needed to verify and extend the role of OPN, to determine whether or how OPN plays a role in the progress of AE, and to confirm the clinical usefulness of OPN as a biomarker for AE-IPF occurrence and predictor of survival in IPF patients.

## Figures and Tables

**Table 1:** Clinical characteristics of patients with IPF.

Clinical characteristics	Controls (*n* = 20)	AE-IPF patients (*n* = 32)	S-IPF patients (*n* = 39)	*p* value (AE-IPF vs S-IPF)
Age (years)	61.05 ± 6.59	69.84 ± 8.28	66.31 ± 7.93	0.097
Male	85% (17)	84% (27)	97% (38)	0.051
Smoker (%)	0 (0)	56% (18)	44% (17)	0.205
Corticosteroids (treated in hospital)	0 (0)	88% (28)	13% (5)	<0.001^*∗∗∗*^
FVC% predicted	NA	57.72 ± 14.23	69.69 ± 16.43	0.016^*∗*^
DLCO% predicted	NA	35.61 ± 11.15	52.05 ± 17.56	0.006^*∗∗*^
LDH (U/L)	NA	390.97 ± 152.74	224.27 ± 36.60	<0.001^*∗∗∗*^
CRP (mg/L)	NA	55.88 ± 55.01	12.05 ± 21.01	<0.001^*∗∗∗*^

^*∗*^ *p* < 0.05; ^*∗∗*^*p* < 0.01; ^*∗∗∗*^*p* < 0.001; *p* values were calculated using the Mann–Whitney test. CRP, C-reactive protein; LDH, lactate dehydrogenase; DLCO, diffusing lung capacity of carbon monoxide; FVC, forced vital capacity; NA, not applicable.

**Table 2:** Predictive value of acute exacerbation in patients with idiopathic pulmonary fibrosis identified by logistic regression analysis.

Variable	Odds ratio	95% CI	*p* value
Age (years)	1.057	0.994–1.123	0.075
Male	7.037	0.777–63.699	0.083
Smoker	0.541	0.210–1.393	0.203
CRP	1.039	1.013–1.064	0.002^*∗∗*^
LDH	1.035	1.017–1.053	<0.001^*∗∗∗*^
FVC% predicted	0.950	0.908–0.993	0.024^*∗*^
DLCO% predicted	0.929	0.878–0.983	0.010^*∗*^
OPN (ng/ml)	1.305	1.087–1.567	0.004^*∗∗*^
KL-6 (U/ml)	1.001	1.000–1.002	0.010^*∗*^

^*∗*^ *p* < 0.05, ^*∗∗*^*p* < 0.01, ^*∗∗∗*^*p* < 0.001. The data are shown as the OR with the 95% CI. OR, odds ratio; CI, confidence interval.

**Table 3:** Ability of OPN to predict mortality in patients with idiopathic pulmonary fibrosis when evaluated by Cox regression.

Variables	Univariate analysis			Multivariate analysis		
	HR	95% CI	*p* value	HR	95% CI	*p* value
Age	1.055	1.007–1.105	0.025^*∗*^	1.055	1.007–1.015	0.024
Male	2.766	0.953–8.032	0.061			
Smoker	0.847	0.398–1.801	0.066			
FVC%	0.961	0.923–1.001	0.057			
DLCO%	0.965	0.926–1.006	0.092			
CRP	1.012	1.006–1.019	<0.001^*∗∗∗*^	1.013	1.013–1.019	<0.001^*∗∗∗*^
LDH	1.009	1.006–1.012	<0.001^*∗∗∗*^	1.009	1.006–1.013	<0.001^*∗∗∗*^
OPN (continuous)	1.100	1.006–1.202	0.036^*∗*^	1.010	1.001–1.019	0.032^*∗*^
KL-6 (continuous)	1.000	1.000–1.000	0.007^*∗∗*^	1.000	1.000–1.000	0.007^*∗∗*^

^*∗*^ *p* < 0.05, ^*∗∗*^*p* < 0.01, ^*∗∗∗*^*p* < 0.001. The data are shown as the HR with the 95% CI. HR, hazard ratio; CI, confidence interval.

## Data Availability

The data used to support the findings of this study are available from the corresponding author upon request.

## Abbreviations

AE: Acute exacerbation
IPF: Idiopathic pulmonary fibrosis
FVC%: Percent forced vital capacity
DLCO%: Percent diffusing capacity
AUCs: Areas under the curve
HRCT: High-resolution computed tomography
SD: Standard deviation
KM: Kaplan–Meier
ROC: Receiver operating characteristic.
